# Long-Term Safety of Transplanting Human Bone Marrow Stromal Cells into the Extravascular Spaces of the Choroid of Rabbits

**DOI:** 10.1155/2017/4061975

**Published:** 2017-06-18

**Authors:** Adi Tzameret, Sapir E. Kalish, Ifat Sher, Lea Twito, Amilia Meir, Itay Levy, Shlomo Margel, Iris Moroz, Mordechai Rosner, Avraham J. Treves, Arnon Nagler, Michael Belkin, Ygal Rotenstreich

**Affiliations:** ^1^Maurice and Gabriela Goldschleger Eye Institute, Sheba Medical Center, Tel Hashomer, Israel; ^2^Sackler School of Medicine, Tel Aviv University, Tel Aviv, Israel; ^3^Center for Stem Cells and Regenerative Medicine, Sheba Medical Center, Tel Hashomer, Israel; ^4^Department of Chemistry, Bar-Ilan Institute of Nanotechnology and Advanced Materials, Ramat Gan 52900, Israel; ^5^Hematology Division, Sheba Medical Center, Tel Hashomer, Israel

## Abstract

Incurable neuroretinal degeneration diseases cause severe vision loss and blindness in millions of patients worldwide. In previous studies, we demonstrated that transplanting human bone marrow stromal cells (hBMSCs) in the extravascular spaces of the choroid (EVSC) of the Royal College of Surgeon rats ameliorated retinal degeneration for up to 5 months. Assessing the safety of hBMSC treatment and graft survival in a large animal is a crucial step before initiating clinical trials. Here, we transplanted hBMSCs into the EVSC compartment of New Zealand White rabbits. No immunosuppressants were used. Transplanted cells were spread across the EVSC covering over 80 percent of the subretinal surface. No cells were detected in the sclera. Cells were retained in the EVSC compartment 10 weeks following transplantation. Spectral domain optical coherence tomography (SD-OCT) and histopathology analysis demonstrated no choroidal hemorrhages, retinal detachment, inflammation, or any untoward pathological reactions in any of transplanted eyes or in the control noninjected contralateral eyes. No reduction in retinal function was recorded by electroretinogram up to 10 weeks following transplantation. This study demonstrates the feasibility and safety of transplanting hBMSCs in the EVSC compartment in a large eye model of rabbits.

## 1. Introduction

Age-related macular degeneration (AMD) is the leading cause of blindness and severe visual impairment in the industrial world, with over 30 million affected people, mostly in older age [1]. The disease is associated with chronic oxidative stress and inflammation that ultimately lead to protein damage, aggregation, and degeneration of retinal pigment epithelium (RPE) cells and concomitant degeneration of the photoreceptor cells. Accumulation of extracellular deposits (drusen) between the RPE and their underlying basement membrane (Bruch's membrane) further accelerates RPE cell death that leads to photoreceptor degeneration (“dry” AMD). Ten to fifteen percent of AMD patients develop neovascular (“wet”) AMD characterized by choroidal neovascularization, invasion of abnormal blood vessels, and fluid leakage into the retina [2]. Retinitis pigmentosa (RP) is the most common cause of hereditary untreatable neuroretinal degeneration. It is a highly genetically heterogeneous disease where an initial loss of rod photoreceptors is followed by degeneration of cone cells and other retinal neurons as well as the RPE [3]. Although there are treatments for “wet” AMD, which decrease new blood vessel formation and improve vision [4], macular and retinal degeneration continue to progress and there is no effective treatment that can stop or slow down the progressive neurodegeneration of retinal cells in dry AMD and RP.

Bone marrow stromal cells (BMSCs), described over 20 years ago, have been extensively studied and have been considered excellent candidates for cell therapy for neuroretinal degeneration diseases. These cells exert paracrine effects by secreting neurotrophic and survival-promoting growth factors and cytokines, as well as protective and reparative micro- and nano- lipid microvesicles [5, 6]. BMSCs express low levels of major histocompatibility complex (MHC) class I molecules and no MHC class II molecules [7] suggesting the possibility of allogeneic transplantation. Moreover, they are immunosuppressive and inhibit the release of proinflammatory cytokines [8–11]. These properties make BMSCs excellent candidates for cell therapy for neuroretinal degeneration diseases.

One of the major challenges in ophthalmic stem cell therapy is the difficulty to safely deliver effective doses of cells in proximity to the entire area of the affected tissue, the retina. Current methods of cell delivery to the eye are insufficient and fraught with considerable risks. Thus, safety problems and the blood–retina barrier limit the use of systemic application. Intravitreal injections are currently used in the clinic for delivery of certain pharmaceuticals (such as antiangiogenic treatments). However, we and others have shown that intravitreal injection is less effective for delivery of stem cells as cells diffuse poorly through the vitreous gel [12]. Better results have been obtained using subretinal injection of stem cells. Subretinal transplantation of hBMSCs was shown to improve retinal function in rat and mouse models of AMD and RP [13–16]. In those studies, cells were transplanted via a transscleral-transchoroidal approach, creating a localized subretinal “bleb”. Limited number of cells could be injected, and photoreceptor rescue was restricted to areas in proximity to the injection site. Hence, this approach may not be efficient in treating AMD or RP, in which large areas of the retina are damaged. The subretinal transplantation involves 3-port pars plana vitrectomy and localized retinal detachment of the treated area which requires multiple sites of injection in each eye to deliver adequate treatment dosage per retinal area [17, 18]. Moreover, the subretinal surgery raises a significant safety issue, as the retinal architecture across the entire retina in AMD and RP patients is fragile and the surgery can induce mechanical damage, reactive gliosis, and loss of function [19–22]. These procedural effects were documented in a recent gene therapy trial in which patients receiving a subretinal injection under the foveal region lost retinal thickness and visual acuity [23], suggesting that subretinal surgery may present a risk in treating the fovea, which is the target tissue in AMD.

To overcome the above indicated limitations of subretinal cell transplantation, we have developed a novel method of cell transplantation into the extravascular spaces of the choroid (EVSC) underlying the retina. In a previous study, we demonstrated that transplantation of hBMSCs in the EVSC of a rat model of AMD and RP resulted in photoreceptor rescue along most of the retina and significantly enhanced retinal function for up to 5 months following cell transplantation. No retinal detachment, hemorrhages, or signs of inflammation were observed even though rats were transplanted with human cells, and no immunosuppression medications were given [12]. This study established the rational for transplanting hBMSCs in the EVSC compartment as a possible treatment for AMD and RP. In subsequent studies, we demonstrated the efficient delivery of hBMSCs into the EVSC of rabbits using a novel injector [24, 25].

Assessments of long-term safety and efficacy of cell transplantation into the EVSC compartment in a large animal model are crucial as a step towards clinical trials. In the present study, we examined the short- and long-term safety of transplanting human cells in the EVSC compartment of rabbits. We focused on the localization of transplanted cell in the eye, retention of transplanted cells, and evidence of untoward pathology.

## 2. Methods

### 2.1. Animals

Fourteen New Zealand White (NZW) rabbits were purchased from Harlan Laboratories, Rehovot, Israel, and kept until surgery at the Sheba Medical Center animal facility. All animal procedures and experiments were conducted with approval and under the supervision of the Institutional Animal Care Committee at the Sheba Medical Center, Tel Hashomer, and conformed to the recommendations of the Association for Research in Vision and Ophthalmology Statement for the Use of Animals in Ophthalmic and Vision Research.

### 2.2. Stem Cell Preparation

Fresh human bone marrows were derived from 3 healthy volunteers (ages 35–50 years). Bone marrow samples were collected in the operating room under sterile conditions, under the approval of the Institutional Review Board at Sheba Medical Center, Tel Hashomer (Research no. 0816-13-SMC). Bone marrow mononuclear cells were separated by Ficoll gradient (1.077 g/dl) according to the manufacturer instructions and were seeded in tissue culture flasks with culture media containing low-glucose Dulbecco's Modified Eagle's Medium (DMEM) supplemented with 15% FCS, 100 U/ml penicillin, 100 *μ*g/ml streptomycin, and 2 mM L-Glutamine. Tissue culture media was changed after 48 h and then twice a week until 70–80% confluence was reached. Cell surface antigen phenotyping was performed on all passages on cells from all 3 donors by flow cytometry (FACSCalibur, Becton-Dickinson) using antibodies directed against CD14, CD34, CD45, CD73, CD90, CD105, and HLA-DR [12]. Cells from all 3 donors expressed the mesenchymal cell surface markers CD73, CD90, and CD105 and were negative for the expression of the hematopoietic markers CD14 and CD34, the surface human leukocyte antigen DR (HLA-DR) and the endothelial cell marker CD31. High cell viability was demonstrated by lack of incorporation of the 7AAD peptide. Proliferation index was tested within the first 72 hours after seeding by XTT reagent according to the manufacturer instructions (Biological industries, Beit Haemek, Israel). Cells from the 3 donors were used for transplantation.

### 2.3. Cell Labeling with 1,1′-Dioctadecyl-3,3,3′,3′-tetramethylindocarbocyanine Perchlorate

The hBMSC were labeled prior to transplantation with Dil (1,1′-dioctadecyl-3,3,3′,3′-tetramethylindocarbocyanine perchlorate) by incubating a cell suspension at a concentration of 1 × 10^6^ cell/mL in 70 *μ*l PBS containing 0.27 mM Dil.

Following 15 min incubation at 37°C, cells were extensively washed with PBS and resuspended in 10% human serum albumin solution (Zenalb® 20, Kamada) to a final concentration of 1 × 10^6^ cells/ml for transplantation.

### 2.4. Cell Transplantation

 Fifteen million hBMSCs were transplanted into the EVSC compartment of one eye in NZW rabbits. Rabbits were under ketamine (35 mg/kg) and xylazine (5 mg/kg) intramuscular anesthesia. Cell transplantation was performed under a surgical microscope (Leica Wild M690; Herring, Switzerland). Following peritomy in the superior temporal quadrant, a 3.0 mm linear tangential incision was preformed 4 mm posterior to the limbus using an AK diamond knife (Accutome, Inc. Malvern, PA). A 3 mm scleral tunnel was generated using a diamond keratome knife (Accutome Inc. Malvern, PA) at an angle of 20° toward the choroid. The injector's pin [26] was extended and inserted into the scleral tunnel to extend it, and 0.3 milliliters of cell suspension solution was injected. The procedure took 3–5 minutes. Rabbits were euthanized with sodium pentobarbital (140 mg/Kg), and the eyes were removed for histology analysis. Rabbits that were kept for follow-up received Dipyrone in drinking water (1gr/L) for 12 hours after the injection.

### 2.5. Spectral Domain Optical Coherence Tomography (SD-OCT)

Rabbits were anesthetized with an intramuscular injection of ketamine (35 mg/kg) and xylazine (5 mg/kg), and their pupils were dilated by topical administration of 1 drop of 0.5% tropicamide eye drops (Fisher Pharmaceuticals Labs, Bnei Brak, Israel) before image acquisition. Corneas were kept moist with 2.5% hydroxypropyl methylcellulose (Fisher Pharmaceuticals Labs, Bnei Brak, Israel). Eyes were subjected to OCT imaging with Spectralis® Multicolor SD-OCT (Heidelberg Engineering, Heidelberg, Germany). Rabbits were placed on a platform with the visual streaks (3 mm ventral to the optic nerve head, ONH) located at the center of the image. We performed rectangular scans with 768 A-scan per B-scan, with a total of 25 B-scan per frame. SD-OCT scans were analyzed using ImageJ (version 1.5d; National Institutes of Health, Bethesda, MD, USA), registering 7 B-scans per image using the Stackreg plug-in [27]. Thickness of the following layers was measured: ganglion cell layer (GCL), inner plexiform layer (IPL), inner nuclear layer (INL), outer plexiform layer (OPL), outer nuclear layer (ONL), RPE, choroid, and sclera. Duration of the procedure was approximately 15 min per eye.

### 2.6. Electroretinogram (ERG) Recording

Rabbits were anesthetized with intramuscular injection of ketamine and xylazine, pupils were dilated with topical 1% tropicamide, and the corneas kept moist with 2.5% hydroxypropyl methylcellulose. ERGs were recorded from both eyes simultaneously using corneal contact lens ERG-jet electrode (Micro Components, Universo Pastique, Switzerland). A chloride silver reference electrode was placed subcutaneously near the temporal canthus. The ground electrode was placed on the ear. Responses were amplified at 10,000 gain at 0.1–1000 Hz, filtered to remove 60 Hz noise, and digitized at a 10 kHz rate. For dark-adapted ERG, rabbits were kept in total darkness for 2 h prior to testing and responses were averaged with stimulus intervals of 1–30 s depending on the stimulus luminance level. For light-adapted ERG, the rabbits were light-adapted for 10 minutes prior to testing and responses were averaged with stimulus intervals of 1 s. Flash intensities were 0.023, 0.25, 2.4, 4.4, and 23.5 cd-s/m^2^.

### 2.7. Immunofluorescence

Eyes were enucleated and immersed in 4% paraformaldehyde (PFA) for 48 h and infiltrated with a solution containing 2% PFA and 5% sucrose for 1 h. Then, eyes were incubated in sucrose solutions (5%, 10%, 12.5%, and 15% sucrose) for 30 min each and followed by incubation in 20% sucrose for 16 h. Eyes were embedded in 20% sucrose in optimum cutting temperature (O.C.T, Sakura Finetek Inc. Torrance, CA, USA). Ten-micrometer cryosections along the vertical meridian of the eye through the optic nerve were cut on a cryostat. For detection of transplanted cells, sections were blocked in PBS containing 5% normal goat serum, 1% BSA, and 0.3% Triton-X-100 for 30 min at room temperature. Then, sections were incubated with anti-human nuclei antibody, clone 3E1.3 (EMD Millipore, Temecula, CA, USA) for 20 hr at 4°C and followed by Alexa-fluor568-labeled goat anti-mouse secondary antibody (Jackson ImmunoResearch, West Grove, PA). For detection of choroidal blood vessels, sections were incubated with monoclonal mouse anti-smooth muscle actin (clone 1A4, Sigma-Aldrich Inc. St. Louis, MO, USA) 1 h at room temperature followed by secondary antibody Alexa Fluor 488-conjugated goat anti-mouse antibody (Jackson ImmunoResearch, West Grove, PA). Sections were counterstained with DAPI (4′,6-diamidino-2-phenylindole, Sigma-Aldrich Inc. St. Louis, MO, USA) for 3 minutes, washed with PBS, and examined by a fluorescent microscope (BX51, Olympus) or a Leica SP5 confocal microscope.

### 2.8. Histopathology

Cryosections were prepared as detailed above and stained with hematoxylin and eosin. Light microscopy evaluation of changes in the eye was performed.

### 2.9. Statistical Analysis

MANOVA analysis was performed using SPSS for windows version 20.0. We conducted a 6 (time in weeks) × 5 (amplitude) × 2 (transplanted versus control) MANOVA with time in weeks and stimuli amplitude defined as within-subjects factors, transplanted versus control serving as a between-subjects factor, and the a- and b-wave recordings serving as dependent variables. Differences were considered significant if *p* < 0.05.

## 3. Results

### 3.1. Transplanted hBMSCs Are Maintained in the EVSC Compartment of Rabbit Eyes for 10 Weeks

In our previous studies, we demonstrated the transplantation of Dil-labeled human stem cells across the EVSC compartment. Transplanted cells were localized in a thin homologue layer, covering 87 ± 3% (mean ± SE) of the subretina surface 2 hours following transplantation [24, 25].

**Figure 1:**
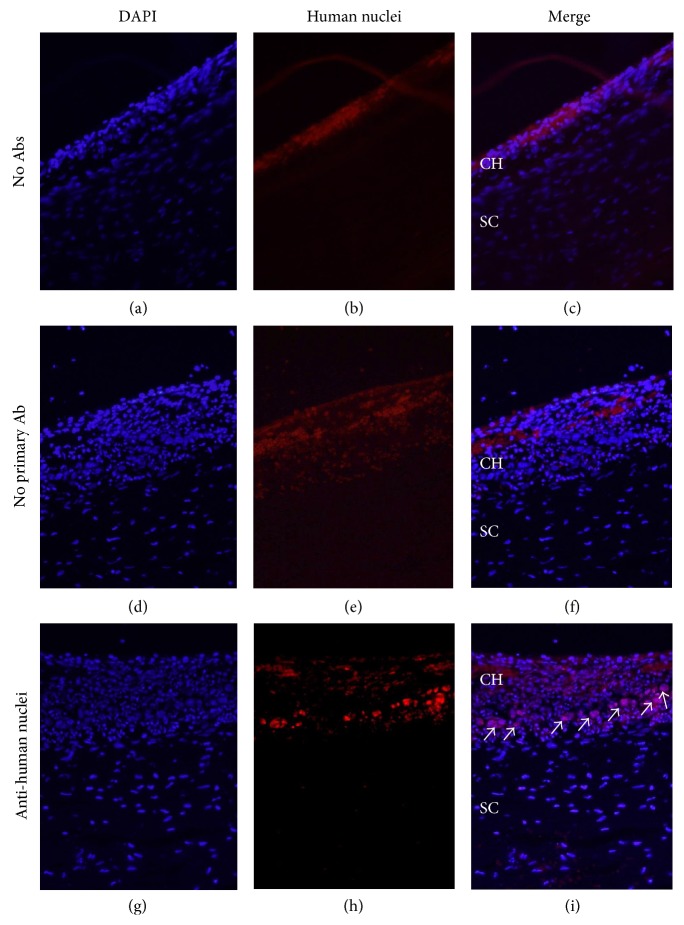
Identification of transplanted hBMSCs in the EVSC compartment 2 hours following transplantation. Frozen sections of rabbit eyes removed 2 hours following transplantation of nonlabeled hBMSCs were stained with anti-human nuclei antibody (red, (h) and (i)) to identify the transplanted human cells. White arrows highlight transplanted cell. Sections counterstained with DAPI only (no antibodies, (a), (b), and (c)) or incubated with secondary antibody only (d), (e), and (f) are presented as control. All sections were counterstained with DAPI (blue). CH: choroid; SC: sclera; Ab: antibody. Scale bar 100 *μ*m.

**Figure 2:**
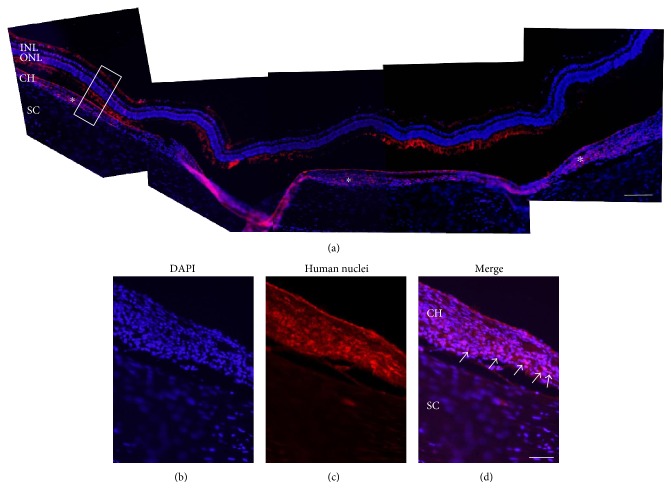
Transplanted hBMSCs are spread throughout the EVSC 2 weeks following transplantation. (a) A mosaic of histological sections of a rabbit eye removed 2 weeks following transplantation of nonlabeled hBMSCs. Cells were identified using anti-human nuclei monoclonal antibody (red), and sections were counterstained with DAPI (blue). White asterisks highlight areas in the EVSC where hBMSCs were identified. Scale bar 50 *μ*m. ((b), (c), and (d)) A large magnification of the indicated area in the image presented in (a). White arrows highlight some of the transplanted cells. Scale bar 100 *μ*m. INL: inner nuclear layer; ONL: outer nuclear layer; CH: choroid; SC: sclera. Control sections without antibody incubation and incubated with secondary antibody as control are presented in Supplementary Figure 1 available online at https://doi.org/10.1155/2017/4061975.

**Figure 3:**
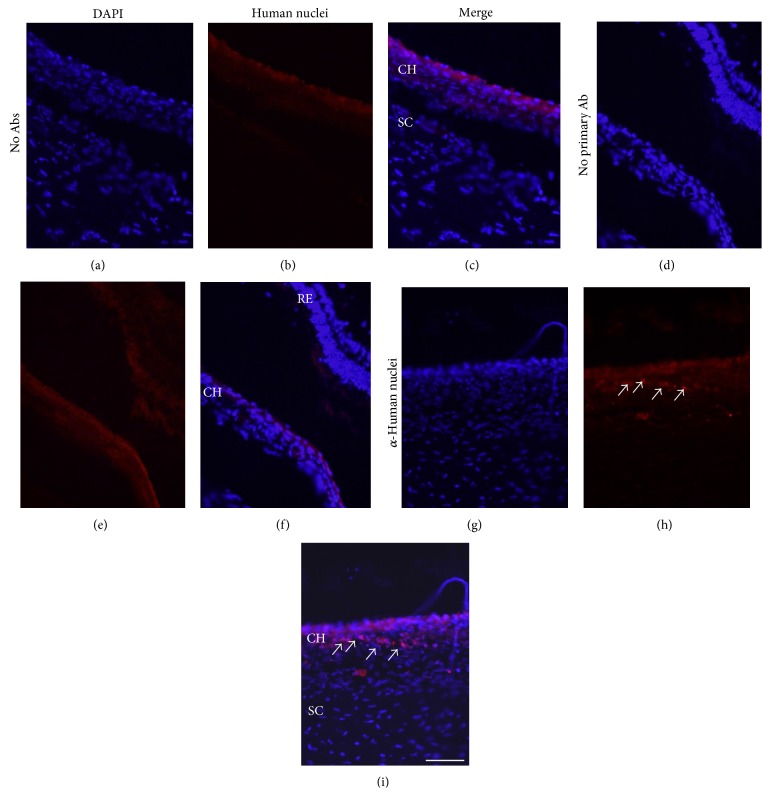
Transplanted hBMSCs identified in the EVSC 10 weeks following transplantation. Frozen sections of rabbit eyes removed 10 weeks following transplantation of nonlabeled hBMSCs were stained with anti-human nuclei antibody (red, (h) and (i)). White arrows highlight some of the transplanted cells. Sections stained with DAPI only (no antibodies, (a), (b), and (c)) or incubated with secondary antibody only (d), (e), and (f) are presented as control. All sections were counterstained with DAPI (blue). Ab: antibody; CH: choroid; SC: sclera; RE: retina. Scale bar 100 *μ*m.

To examine the retention of transplanted cells in the eye, nonlabeled hBMSCs were transplanted in the EVSCs and retinal sections were stained with an anti-human nuclei antibody. As shown in Figures 1–3, transplanted human cells were demonstrated in the EVSC compartment at up to 10 weeks following transplantation. A mosaic of histological sections of a rabbit eye removed two weeks following transplantation of nonlabeled hBMSCs stained with the anti-human antibody identified transplanted cells across the ECSV covering 80 ± 2% (mean ± SE) of the surface of the back of the eye (Figure 2). At time point of 10 weeks, cells were identified in several isolated locations (Figure 3(f)). There was no positive staining of human cells in the sclera of transplanted eyes or in contralateral noninjected control eyes in any of the rabbits (Figures 1–3 and data not shown).

### 3.2. Transplanted hBMSCs Are Located between the Choroidal Blood Vessels

**Figure 4:**
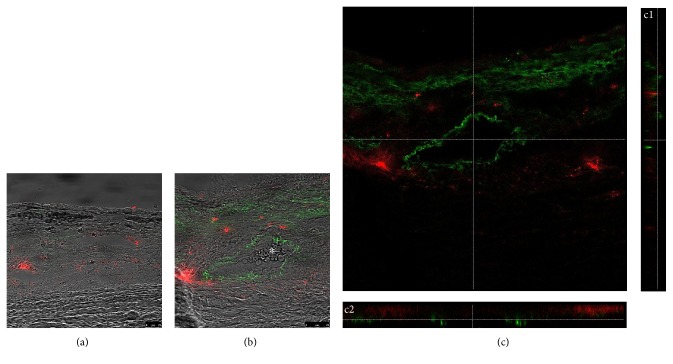
Transplanted hBMSCs are located between the blood vessels in the EVSC. Cross sections of eyes removed 4 days following transplantation of Dil-labeled hBMSCs (red) were stained with an antibody directed against smooth muscle actin (green, (b) and (c)) and photographed using confocal microscopy. (a) The section was incubated without a primary antibody. (b) Dil-labeled cells are clearly identified across the ECVS. The white asterisk highlights red blood cells inside a blood vessel. Scale bar 25 *μ*m. (c) A representative confocal image with orthogonal views (c1 and c2) demonstrating the absence of Dil-labeled cells inside the blood vessel.

To determine the exact localization of transplanted human bone marrow stromal cells within the EVSC compartment, we transplanted Dil-labeled hBMSCs and examined their localization in the EVSC by staining the sections with an antibody directed against smooth muscle actin that is specifically expressed in the smooth muscle cells of the tunica media of the choroidal vessels as well as in extravascular smooth muscle cells that are oriented longitudinally along the external surface of the vessel walls [28].  Figure 4() and Supplementary Figure 2 demonstrate the localization of the transplanted Dil-labeled human BMSCs (red) in the EVSC surrounding the blood vessels 4 days following cell transplantation. No cells were identified within the blood vessels in any of the sections examined.

### 3.3. Histopathology Analysis of Eyes Transplanted with hBMSCs

**Figure 5:**
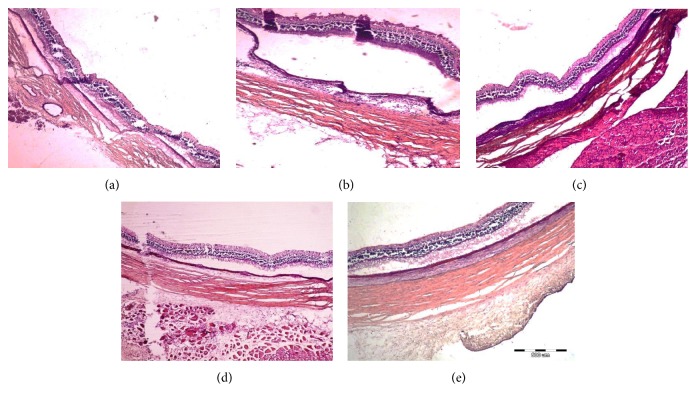
Histopathology analysis of transplanted eyes. Representative images of frozen sections of rabbit eyes removed from control nontransplanted eyes (a) or eyes removed 2 hours (b), 4 days (c), 2 weeks (d), or 10 weeks (e) following transplantation of hBMSCs were stained with hematoxylin-eosin. No inflammatory cells or hemorrhages were detected. Scale bar 500 *μ*m.

Histopathological examination of eyes transplanted with hBMSCs revealed no pathological changes in the retina, choroid, or sclera of transplanted eyes at any time point following transplantation (Figure 5()) or in control contralateral nontransplanted eyes (data not shown). Importantly, no infiltration of lymphocytes or other indications of inflammation were observed in the injected or contralateral eyes.

### 3.4. Evaluation of Cell Transplantation Safety by SD-OCT Imaging

**Figure 6:**
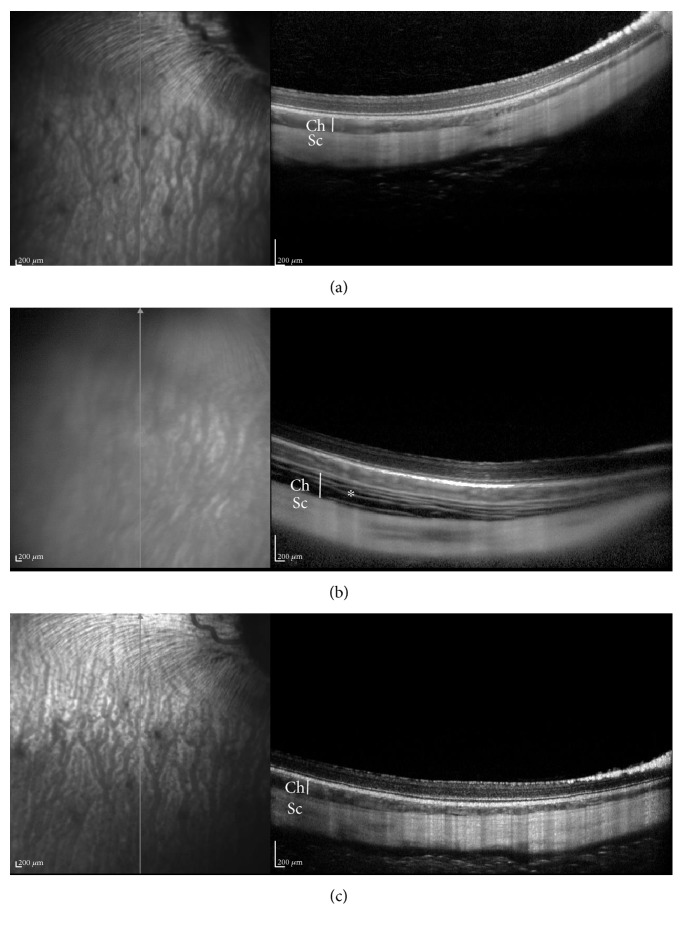
SD-OCT imaging reveals no retinal detachment or choroidal hemorrhages following hBMSC transplantation. SD-OCT images of rabbit eyes before (a), 1 hour (b), and 10 weeks (c) following hBMSC transplantation. Choroidal “stretching” (highlighted with an asterisk) is evident 1 hour following cell transplantation. Choroidal thickness in each time point is highlighted with a white vertical line. Ch: choroid; Sc: sclera.

The safety of cell transplantation was evaluated by SD-OCT imaging. As shown in Figure 6, no retinal detachment or choroidal hemorrhages were demonstrated 1 hour following transplantation (Figure 6(b)). The only irregularity noticeable in the images taken immediately following cell transplantation was “stretching” of the choroid (highlighted with an asterisk and a vertical white line, Figure 6(b)). Choroidal thickness was significantly higher 1 hour following transplantation (92.7 ± 8.7 *μ*m versus 236.8 ± 16.5 *μ*m, mean ± SE, *t*-test *P* value =0.006). At 10 weeks following transplantation, choroidal thickness was not significantly different from baseline (104.9 ± 13.2 *μ*m, *p* = 0.5) and was significantly thinner as compared with 1 hour following transplantation (*p* = 0.009). There were no significant differences in retinal thickness or thickness of the inner retinal layers between different time points (all *p* > 0.05, Table 1).

### 3.5. Evaluation of Retinal Function following Cell Transplantation

**Figure 7:**
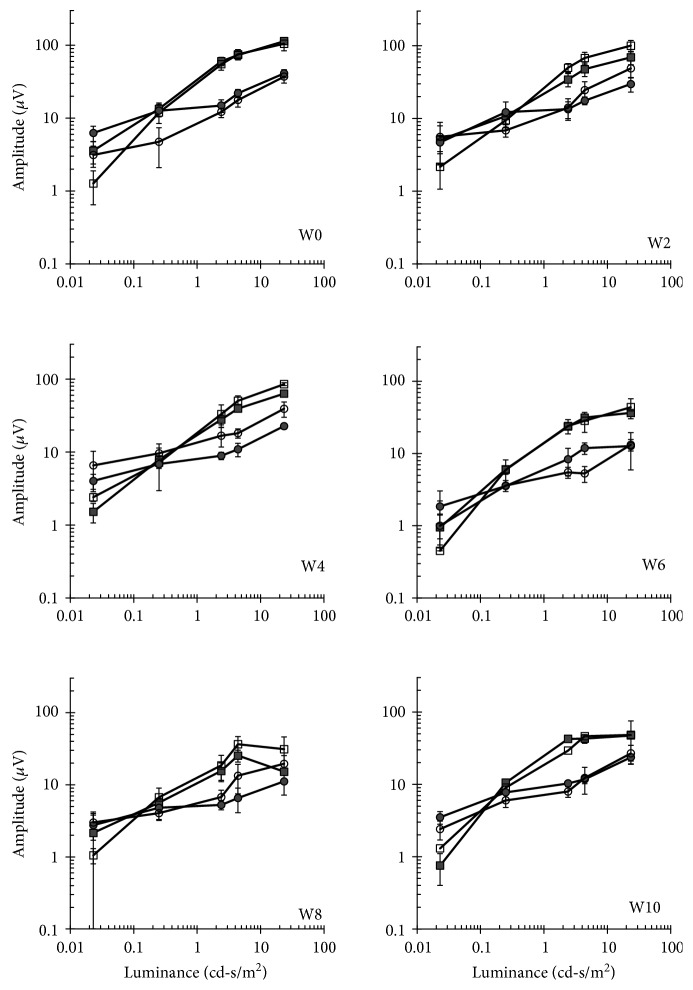
Long-term follow-up of photoreceptor function by a-wave ERG analysis. Mean maximal a-wave amplitude ERG responses to light flashes in increasing luminance levels were recorded following dark (squares) or light (circle) adaptation, in transplanted (closed) or control (open) eyes at indicated weeks (W) prior to (W0) or 2–10 weeks following cell transplantation. Data are presented as mean ± SE.

ERG analysis measures the mass electrical response of the retina to light stimulation and is widely used for objective determination of the functional status of the retina and as an objective safety outcome of different ocular therapies. In our previous study in RCS rats, we demonstrated a transient reduction in ERG in the first week following hBMSC transplantation. At later time points, ERG of transplanted eyes demonstrated improved retinal function in transplanted eyes versus nontransplanted eyes that underwent retinal degeneration as expected by the course of retinal degeneration in this rat model [12]. In addition, in clinical trials aimed at determination of safety of encapsulated cell implant for delivery of ciliary neurotrophic factor, a reduction in the amplitude of the scotopic b-wave was observed in 4 out of 7 participants due to implantation [29]. To examine the effect of hBMSC transplantation on retinal function in the rabbits, nonlabeled cells were transplanted into the right eye of 3 rabbits and ERG analysis was performed in both eyes simultaneously prior to and every 2 weeks following cell transplantation. Specifically, we measured the amplitudes of the b-wave which is generated by the bipolar cells of the inner retina and is most commonly used parameter in clinical studies and animal research and the a-wave which is generated by the cones and rods in the outer photoreceptor layer [30–32]. The responses of cones and rods can be isolated when the test is performed under light or dark adaptation, respectively [32, 33]. No significant differences were observed between the injected and control, noninjected contralateral eyes at any of the time points tested (Figures 7() and 8()).

## 4. Discussion

Transplantation of hBMSCs is a promising potential therapy for neuroretinal degeneration. Current surgical transplantation methods are limited by the highly invasive injection procedure and the small amount of cells that can be injected necessitating multiple injections to prevent retinal detachment [17, 18].

The new injection system used in this study enables minimally invasive and safe delivery of cell therapy from the same location used for intravitreal injections directly to the target tissues (sub-RPE and extravascular spaces of the choroid). Large volumes (300 *μ*l, 15 × 10^6^ cells) can be injected without insertion of any surgical tools near the central retina, penetration of the retina, or choroid. The new method does not require three-port pars plana vitrectomy, subretinal bleb formation, or multiple injections that are currently being used in clinical trials for delivery of stem cell-derived retinal pigment epithelium cells in close proximity to the RPE [17, 18]. In our previous study, we demonstrated that cells are spread under most of the retina of rabbits (87% of the posterior segment surface) including the central retina 1 hour following cell injection [24, 25]. Here, we demonstrated that transplanted cells were retained across the EVSC covering 80% of the posterior surface for two weeks after transplantation. At 10 weeks following transplantation, some cells were retained in the EVSC at several isolated locations. In our previous study in the RCS rats, transplanted hBMSCs were retained in the EVSCs only for 2 weeks, but photoreceptor degeneration was ameliorated for up to 22 weeks following transplantation. Hence, our findings that hBMSCs are retained in the large eye model of rabbits for a longer duration are very promising for future clinical use in patients. Using both Dil labeling and antibody staining, we did not detect any human cells in the sclera.

Following cell transplantation, an increase in choroidal thickness was observed using SD-OCT 1 hour after cell transplantation. Interestingly, after injection of a similar volume of dyes and narrow-size iron oxide nanoparticles into the EVSC of rabbit eyes, there was no significant change in choroidal thickness [26]. These findings may suggest that the viscosity of injected solution may have an effect on choroidal thickness following injection. Future experiments will be aimed at testing this possibility, including injection of the cells in a different carrier solutions and different cell concentration as well as other injection parameters (e.g., injection rate, injected volume). Nevertheless, the functional imaging and histopathology analyses did not reveal any ischemic or hemorrhagic findings.

Some transplanted human cells were retained in the EVSC of rabbit eyes for 10 weeks following transplantation, without the need for immunosuppression with no indication of hemorrhages or inflammation. In previous studies, we demonstrated that IONP-delivery of FGF2 can enhance hBMSC growth and differentiation in vitro [34]. In future studies, we will examine the possibility of prolonging graft survival in the EVSC by coinjection with IONPs loaded with growth factors that promote hBMSC survival.

This long-term 10-week follow-up demonstrated no pathological changes in retinal structure or function in any of rabbit eyes xenografted with hBMSC, by imaging (SD-OCT), histopathology analysis, and ERG functional analysis. Moreover, as the eye is considered immune-privileged and since hBMSCs are immunosuppressive and present low levels of antigen presenting MHCI and MHCII, our current and previous [12] studies suggest that allogeneic transplantation of these cells using our method in the EVSC compartment is predicted to be safe in patients with no need for immunosuppression. Furthermore, as the therapeutic effect of xenograft transplantation of human BMSCs in rats was maintained for 5 months [12], allogeneic transplantation of hBMSCs has the potential to promote photoreceptor rescue in patients for several months.

## 5. Conclusions

In this study, we demonstrated the long-term safety of transplanting hBMSCs in the EVSC compartment of a large eye animal model, using a minimally invasive and simple surgical procedure as a crucial step before proceeding into clinical trials. This method may improve the efficacy and safety of other stem cell-based therapies, gene therapies, and possibly other posterior segment treatments.

## Supplementary Material

Supplementary Figure 1. Identification of hBMSCs in the EVSC 2 weeks following transplantation. Frozen sections of rabbit eyes removed at 2 weeks following transplantation of hBMSCs were stained with DAPI only (with no antibodies, panels A-C) or incubated with secondary antibody only (D-F). All sections were counter-stained with DAPI (blue). The lack of nuclear staining in the EVSC demonstrates the specific staining of the anti human nuclei antibody shown in Figure 2. Scale bar 100µm. Ab- antibody. Supplementary Figure 2. Transplanted hBMSCs are located between the blood vessels in the EVSC compartment. Frozen sections of eyes removed 4 days following transplantation of DiI-labeled hBMSCs (red, B & D) were stained with an antibody directed against smooth muscle actin (SMA, green, C & D) and photographed using a fluorescent microscope. Sections were counter-stained with DAPI (blue, A &D). Scale bar 100 µm. Supplementary Figure 3. Representative fluorescent images of a non-transplanted rabbit eye demonstrating the tissue autofluorescence. Frozen sections of non-transplanted eyes were stained with DAPI and photographed with a fluorescent microscope using a similar exposure time (200 msec) used for photographing the anti-human nuclei antibody staining of transplanted eyes. ONL – outer nuclear layer; OS – outer segments; CH- choroid; SC-sclera. Scale bar 100 µm.

## Figures and Tables

**Figure 8:**
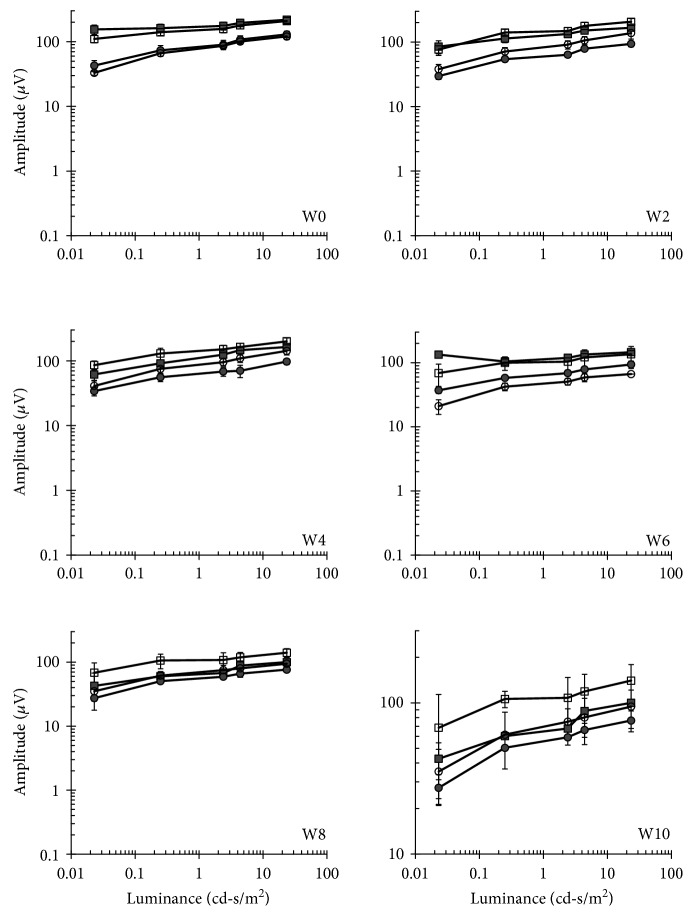
Long-term follow-up of outer retinal function by b-wave ERG analysis. Mean maximal b-wave amplitude ERG responses to light flashes in increasing luminance levels were recorded following dark (squares) or light (circle) adaptation, in transplanted (closed) or control (open) eyes at indicated weeks (W) prior to (W0) or 2–10 weeks following cell transplantation. Data are presented as mean ± SE.

**Table 1:** Mean retinal thickness in rabbit eyes before and following hBM-MSC transplantation.

	Total retina	RNFL	GCL	IPL	INL	OPL	ONL	RPE	ORL	Choroid	Sclera
Baseline	182.5 ± 0.5	4 ± 1	6.8 ± 1.5	11.6 ± 0.0	6 ± 0.6	14 ± 1.3	61.5 ± 0.8	12.6 ± 1	65.8 ± 0.5	92.7 ± 8.7	232 ± 3.2
1 h	195.3 ± 3.7 (p_0_ = 0.07)	6 ± 2.5 (p_0_ = 0.6)	7.6 ± 1.9 (p_0_ = 0.8)	11.7 ± 0.7 (p_0_ = 0.9)	6.8 ± 1.2 (p_0_ = 0.7)	15.9 ± 4.4 (p_0_ = 0.8)	64.5 ± 3 (p_0_ = 0.5)	14 ± 0.9 (p_0_ = 0.5)	68.3 ± 0.7 (p_0_ = 0.3)	236.8 ± 15.6 (p_0_ = 0.006)	243.8 ± 11.1 (p_0_ = 0.5)
10 w	166.7 ± 16 (p_0_ = 0.43, p_1_ = 0.11)	8.9 ± 3.9 (p_0_ = 0.34, p_1_ = 0.6)	8.3 ± 1.3 (p_0_ = 0.54, p_1_ = 0.84)	16.4 ± 4.7 (p_0_ = 0.42, p_1_ = 0.29)	9.2 ± 2.2 (p_0_ = 0.3, p_1_ = 0.4)	12.5 ± 2.8 (p_0_ = 0.7, p_1_ = 0.6)	42.1 ± 18 (p_0_ = 0.4, p_1_ = 0.2)	12.1 ± 0.5 (p_0_ = 0.7, p_1_ = 0.2)	62.8 ± 1.5 (p_0_ = 0.2, p_1_ = 0.07)	104.9 ± 13.2 (p_0_ = 0.5, p_1_ = 0.009)	259.9 ± 1.2 (p_0_ = 0.2, p_1_ = 0.7)

Comparison of retinal thickness measurements (in *μ*m) using SD-OCT in rabbit eyes before (baseline), 1 hour (1 h), and 10 weeks (10 w) following hBMSC transplantation. All data are presented as mean ± SE from 4 different areas from 2 (baseline, 10 weeks) or 3 (1 hour) rabbits. Scleral thickness was measured from the outer scleral border to the choroid/sclera interface. Student's *t*-test was used to evaluate differences in retinal thickness measurements between baseline and following transplantation (p_0_) and between 1 hour and 10 weeks (p_1_) following transplantation. RNFL: retina nerve fiber layer; GCL: ganglion cell layer; IPL: inner plexiform layer; INL: inner nuclear layer; OPL: outer plexiform layer; ONL: outer nuclear layer; RPE: retinal pigment epithelium; ORL: outer retina layer. Total retina was measured from RNFL to ORL.

## Abbreviations

AMD: Age-related macular degeneration
DAPI: 4′,6-Diamidino-2-phenylindole
Dil: 1,1′-Dioctadecyl-3,3,3′,3′-tetramethylindocarbocyanine perchlorate
ERG: Electroretinogram
EVSC: Extravascular spaces of the choroid
hBMSCs: Human bone marrow stromal cells
IONP: Iron oxide nanoparticles
PFA: Paraformaldehyde
RP: Retinitis pigmentosa
RPE: Retinal pigment epithelium
SD-OCT: Spectral domain optical coherence tomography.

## Additional Points

This work was performed in partial fulfillment of the requirements for a M.Sc. degree by Sapir E. Kalish at the Sackler Faculty of Medicine, Tel Aviv University, Israel.
