# Supplementary material for: Long-Term Safety of Transplanting Human Bone Marrow Stromal Cells into the Extravascular Spaces of the Choroid of Rabbits
Source: Stem Cells Int. 2017 Jun 18;2017:4061975. doi: 10.1155/2017/4061975 (PMC5494107; doi:10.1155/2017/4061975)

Supplementary Figure 1 – Identification of hBMSCs in the EVSC 2 weeks following transplantation.

Frozen sections of rabbit eyes removed at 2 weeks following transplantation of hBMSCs were stained with DAPI only (with no antibodies, panels A-C) or incubated with secondary antibody only (D-F). All sections were counter-stained with DAPI (blue). The lack of nuclear staining in the EVSC demonstrates the specific staining of the anti human nuclei antibody shown in Figure 2. Scale bar 100μm. Ab-antibody.

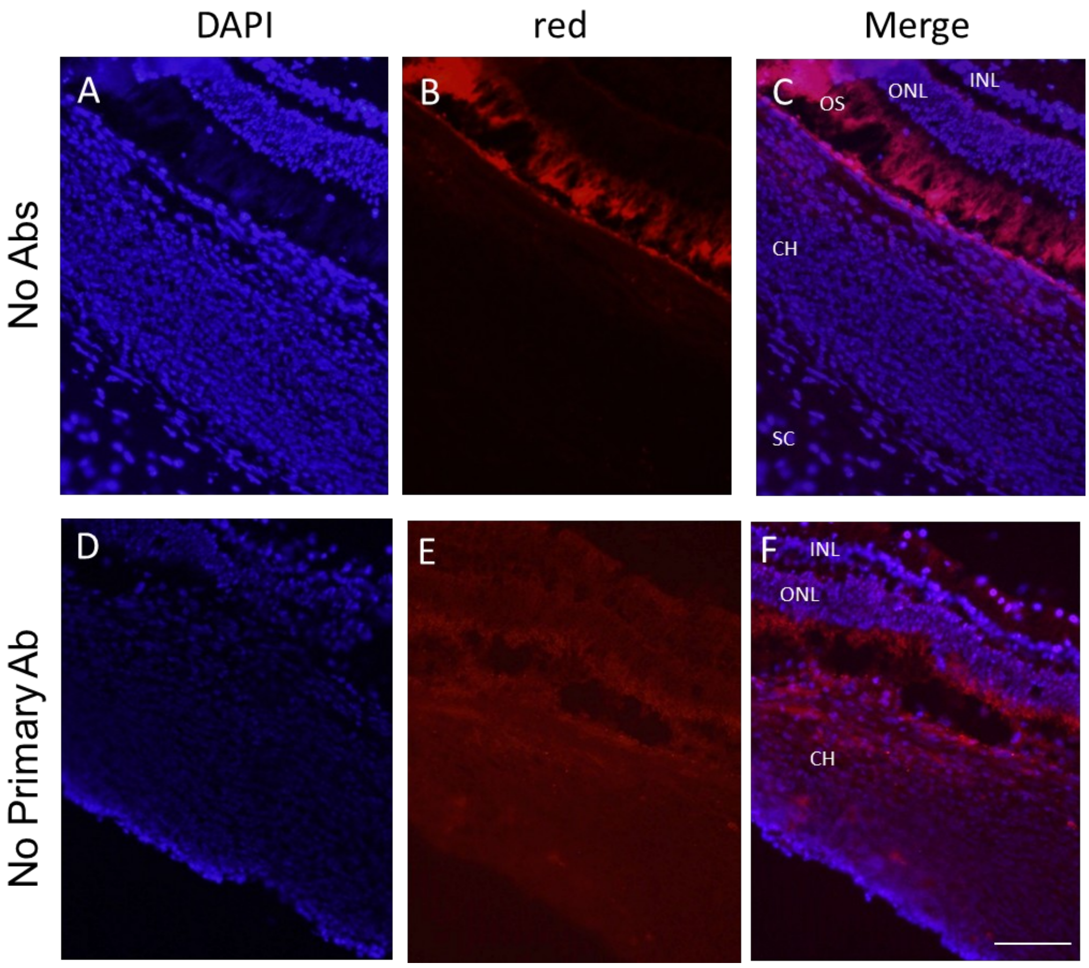

Supplementary Figure 2. Transplanted hBMSCs are located between the blood vessels in the EVSC compartment.

Frozen sections of eyes removed 4 days following transplantation of DiI-labeled hBMSCs (red, B & D) were stained with an antibody directed against smooth muscle actin (SMA, green, C & D) and photographed using a fluorescent microscope. Sections were counter-stained with DAPI (blue, A &D). Scale bar 100 µm.

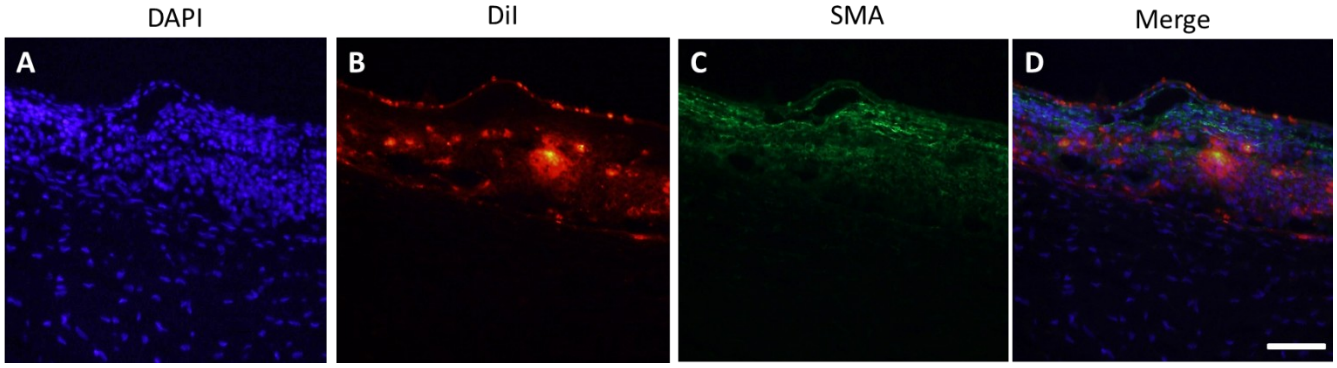

Supplementary Figure 3. Representative fluorescent images of a non-transplanted rabbit eye demonstrating the tissue autofluorescence.

Frozen sections of non-transplanted eyes were stained with DAPI and photographed with a fluorescent microscope using a similar exposure time (200 msec) used for photographing the anti-human nuclei antibody staining of transplanted eyes. ONL – outer nuclear layer; OS – outer segments; CH- choroid; SC-sclera. Scale bar 100 μm.

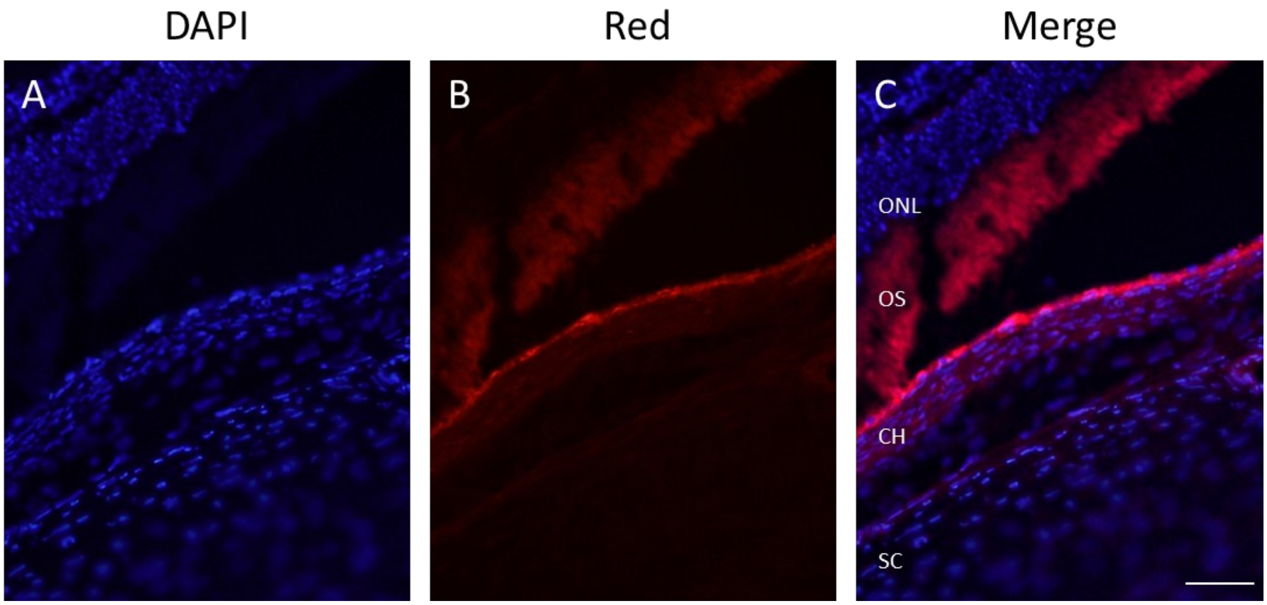

Supplement: Supplementary file 1 — Supplementary Figure 1. Identification of hBMSCs in the EVSC 2 weeks following transplantation. Frozen sections of rabbit eyes removed at 2 weeks following transplantation of hBMSCs were stained with DAPI only (with no antibodies, panels A-C) or incubated with secondary antibody only (D-F). All sections were counter-stained with DAPI (blue). The lack of nuclear staining in the EVSC demonstrates the specific staining of the anti human nuclei antibody shown in Figure 2. Scale bar 100µm. Ab- antibody. Supplementary Figure 2. Transplanted hBMSCs are located between the blood vessels in the EVSC compartment. Frozen sections of eyes removed 4 days following transplantation of DiI-labeled hBMSCs (red, B & D) were stained with an antibody directed against smooth muscle actin (SMA, green, C & D) and photographed using a fluorescent microscope. Sections were counter-stained with DAPI (blue, A &D). Scale bar 100 µm. Supplementary Figure 3. Representative fluorescent images of a non-transplanted rabbit eye demonstrating the tissue autofluorescence. Frozen sections of non-transplanted eyes were stained with DAPI and photographed with a fluorescent microscope using a similar exposure time (200 msec) used for photographing the anti-human nuclei antibody staining of transplanted eyes. ONL – outer nuclear layer; OS – outer segments; CH- choroid; SC-sclera. Scale bar 100 µm. [file 4061975.f1.pdf]
